# Zoonotic and reverse zoonotic transmission of viruses between humans and pigs

**DOI:** 10.1111/apm.13178

**Published:** 2021-10-18

**Authors:** Helena Aagaard Glud, Sophie George, Kerstin Skovgaard, Lars Erik Larsen

**Affiliations:** ^1^ Department of Biotechnology and Biomedicine Technical University of Denmark Kongens Lyngby Denmark; ^2^ Department of Veterinary and Animal Sciences University of Copenhagen Copenhagen Denmark

**Keywords:** Zoonosis, reverse zoonosis, viruses, pig, human

## Abstract

Humans and pigs share a close contact relationship, similar biological traits, and one of the highest estimated number of viruses compared to other mammalian species. The contribution and directionality of viral exchange between humans and pigs remain unclear for some of these viruses, but their transmission routes are important to characterize in order to prevent outbreaks of disease in both host species. This review collects and assesses the evidence to determine the likely transmission route of 27 viruses between humans and pigs.

## INTRODUCTION

Viruses circulating in wildlife reservoirs can spillover into susceptible human populations and contribute significantly to the global burden of human infectious diseases, which cause approximately 2.5 billion infections and 2.7 million deaths each year [1, 2]. Before emerging as zoonotic human pathogens, wildlife‐adapted viruses must first overcome a series of epidemiological barriers, such as behavioral barriers (level of human exposure to zoonotic viruses), interspecies barrier, and immunological barriers [3].

Livestock are able to facilitate viral spillover from wildlife to humans by acting as “epidemiological bridges” or intermediate hosts in the transmission chain [4, 5]. Unsurprisingly, through thousands of years of close contact animal husbandry and intensive farming in recent decades, domesticated animals harbor eight times more zoonotic viruses than predicted in other non‐domesticated mammalian species [6]. Opportunities for viral zoonosis accompany the expansion of human agricultural activities, which provoked over 50% of zoonotic emerging infectious disease (EID) events during the past 70 years [7]. Wildlife, however, is not the only threat to livestock; close contact humans can also be a source of viral zoonosis (hereafter referred to as reverse zoonosis and also known as zooanthroponosis and anthroponosis), which is somewhat understudied [8].

A recent study estimated that humans exchange the highest number of viruses with domesticated pigs (*Sus scrofa domesticus*) (n ≈ 31 viruses), cattle (n ≈ 31 viruses), horses (n ≈ 31 viruses), and dogs (n ≈ 27 viruses), surpassing both domestic cats (n ≈ 16 viruses) and goats (n ≈ 22 viruses) [6]. Pigs have served as intermediate, amplification, and “mixing” hosts in past human epidemics and pandemics (e.g., Japanese encephalitis [9], Nipah [10], and influenza A viruses [11]), and humans have spread viruses to pigs in return (e.g., influenza A virus [12]). Global demand for pork continues to rise and, although pig farming practices differ worldwide, the movement of swine and multiple contact points with humans, i.e., at farms, breeding facilities, slaughterhouses, wet markets, and trade shows, intensifies the opportunities for viral transmission [13, 14, 15]. Furthermore, pigs are increasingly used for xenotransplantation and as animal models for human diseases and conditions due to their physiological, genetic, and immunological similarities to humans [16, 17, 18, 19]. Therefore, understanding the viral exchange at the swine–human interface can help prevent zoonotic and reverse zoonotic viral outbreaks, leading to disease, deaths, culling of swine herds, and economic losses [20].

Predicting EIDs in humans and pigs is challenging. Viral zoonoses are considered rare in humans relative to the extensive viral diversity in the animal kingdom, and viral dynamics are strongly amenable to selection mechanisms resulting in rapid changes to viral landscapes [21, 22, 23, 24]. Spillover events can occur incidentally into “dead‐end” hosts, or viral outbreaks can ensue with sustained onward transmission within the novel host population, and can even become a persistent endemic threat [23, 25]. Determining the natural reservoir species and intermediate hosts of EIDs after a spillover event is also demanding when routine surveillance is not in place [26]. Furthermore, the novel host of an EID can become a newfound viral reservoir and spillover into the next susceptible species, e.g., SARS‐CoV‐2 transmission chain from horseshoe bats‐to‐unknown mammalian intermediate‐to‐humans‐to‐mink‐to‐humans [26, 27, 28].

In this review, we collect genetic‐, pathogenic‐, and immunological‐based evidence to determine the likely direction of viral transmission between humans and pigs with the purpose of identifying viral threats to human and pig health, and the roles humans and pigs play as direct viral reservoirs for each other.

## MATERIALS AND METHODS

A framework of factors (Table S1) was designed and applied in scientific literature surveys to assess the infectivity and transmissibility of 27 viruses naturally found in humans and pigs within the past 70 years. The focus is largely on the detection of human or pig‐associated viruses in the secondary host, genetic variation between viral strains isolated from the two hosts, viral entry into target host cells, detection of viral shedding that indicates viral replication in the host and transmission potential, viral dissemination in the host, and the ability for the host’s immune system to suppress infection. This information is highlighted in Table S2 with distinctions drawn between humans and pigs where appropriate. The viruses were then determined to demonstrate zoonotic, reverse zoonotic, or bidirectional viral transmission according to the definitions in Box 1, and the results are summarized in Table 1.

**Table 1:** Summary of transmission routes and sources of the 27 reviewed viruses.

Virus and taxonomy	Transmission route (→ denotes direction)	Significant viral reservoir
Zoonotic viruses (1): Pigs as major sources of viruses		
Eastern equine encephalitis (EEEV); *Alphavirus; Togaviridae*.	Mosquito (*Aedes, Coquillettidia,* and *Uranotaenia* species) → human/pig [143]: vector‐borne. Pig → mosquito: vector‐borne [43]. Pig → pig/human: oronasal contact with infected oropharyngeal secretions or fecal‐oral [43].	Birds are natural hosts (e.g., wading birds, passerine songbirds, and starlings) [143]. Pigs are potential amplification hosts [43].
Japanese encephalitis (JEV); *Flavivirus; Flaviviridae*.	Mosquito (*Culex* and *Aedes* species) → human/pig: vector‐borne [143]. Pig → mosquito: viremia, vector‐borne [44, 45]. Pig → human: oronasal contact with infected oronasal secretions oronasal secretions [47]. Mosquito → mosquito: transovarial [9].	Aquatic birds are natural hosts. Pigs are amplification hosts [9].
Menangle (MenPV); *Rubulavirus; Paramyxoviridae*.	Fruit bat (*Pteropus* species) → pig: oronasal contact with environmental contamination [59, 62]. Pig → pig: fecal‐oral or urinary‐oral or transplacental [144, 145]. Pig → human: possibly infected bodily fluid in cuts [60].	Fruit bats (*Pteropus* species) are natural hosts [59, 62]. Pigs are possible intermediate hosts [60, 61].
Nipah (NiV); *Henipavirus; Paramyxoviridae*.	NiV‐Malaysia: Fruit bat (*Pteropus* species) → pig: oronasal contact with environmental contamination [146]. Pig → pig: airborne or oronasal contact with infected oronasal secretions [147]. Pig → human: airborne or oronasal contact with infected oronasal secretions [148]. NiV‐Bangladesh: Fruit bat (*Pteropus* species) → human: food‐borne consumption of contaminated date palm sap [149]. Human → human: oronasal contact with infected human bodily fluids, limited transmission chain but caused ˜50% of cases [149]. Pig → human: undocumented but possible [150].	Fruit bats (*Pteropus* species) [151, 152]. Pigs are amplifications hosts for NiV‐Malaysia and potentially for NiV‐Bangladesh [10, 150].
Reston ebola (RESTV); *Ebolavirus; Filoviridae*.	Fruit bat (likely *Miniopterus* species) → pig: oronasal contact with environmental contamination [153]. Pig → pig: oronasal contact with infected nasopharyngeal secretions [58]. Pig → human: oronasal contact with infected nasopharyngeal secretions [58, 154].	Fruit bats (likely *Miniopterus* species) are natural hosts [153]. Pigs are intermediate hosts [154].
Tioman (TioV); *Rubulavirus; Paramyxoviridae*.	Fruit bat (*Pteropus* species) → pig/humans: oronasal contact with environmental contamination [64]. Pig → pig/human: possible airborne or oronasal contact with oronasal secretions [31].	Fruit bats (*Pteropus* species) are natural hosts [31, 63]. Pigs are potentially intermediate hosts [64].
Vesicular stomatitis (VSV); *Vesiculovirus; Rhabdoviridae*.	Vertebrate reservoir → biting insect: vector (biological and mechanical [50, 155]). Biting insect → pig/human: vector. Pig → pig/human: possible vector [46, 50], airborne, oronasal contact with infected oronasal secretions, or contact with infected vesicular lesions [48, 49, 50].	Unknown vertebrate reservoir host but likely multiple livestock (including pigs) and wildlife species [156].
Zoonotic viruses (2): Pigs as minor sources of viruses		
Banna (BAV); *Seadornavirus; Reoviridae*.	Mosquito (*Culex* and *Aedes* species) → human/pig: vector‐borne [157, 158].	Potentially mosquito as replication has been demonstrated in mosquito cell line (C6/36) and replication in mammalian cell lines is not possible (BHK‐21 and Vero) [159]. Although replication in mice has been demonstrated (develop viremia), re‐infection was not possible [160].
Cache Valley (CVV); *Orthobunyavirus; Bunyaviridae*.	Mosquito (*Aedes, Coquillettidia, Culex, Culiseta, Orthopodomyia, Psorophora,* and *Uranotaenia* species) → human/pig: vector‐borne [161, 162]. Mosquito → mosquito: transovarial demonstrated experimentally [163].	Deer [164, 165].
Chandipura (CHPV); *Vesiculovirus; Rhabdoviridae*.	Sandfly (*Phlebotomine*) → human/pig: vector‐borne (demonstrated in mice [166]). Sandfly → sandfly: transovarial and venereal [167].	Potentially sandfly (*Phlebotomine*) species as replication has been demonstrated in vector [166].
Encephalomyocarditis (EMCV); *Cardiovirus; Picornaviridae*.	Rodent → human/pig: fecal/urinal‐oral [168]. Pig → pig: fecal‐oral or oronasal contact with infected nasal secretions [169].	Rodents [169].
Foot‐and‐mouth disease (FMDV); *Aphthovirus; Picornaviridae*.	Pig → pig: airborne, oronasal contact with infected oronasal secretions, physical contact with secretions in cuts, environmental contamination (equipment, clothing, animal feed) [170]. Pig → human: potentially by direct contact with secretions through damaged skin [171, 172].	African Cape buffalo (*Syncerus caffer*) (serotypes SAT‐1, 2, and 3) [173].
Getah (GETV); *Alphavirus; Togaviridae*.	Mosquito (*Culex, Anopheles, Aedes, Armigeres,* and *Mansonia* species) → human/pig: vector‐borne [174]. Pig → pig: vertically to fetus during early stage of pregnancy [175].	Potentially cattle (strong serological evidence) [174].
Louping ill (LIV); *Flavivirus; Flaviviridae*.	Tick (*Ixodes ricinus*) → human/pig: vector‐borne [176, 177]. Sheep → human: contact with infected sheep, sheep tissues, or raw milk [176, 177, 178].	Ticks (*Ixodes ricinus*), sheep, and red grouse [176, 177].
Rabies (RABV); *Lyssavirus; Rhabdoviridae*.	Canine (*Carnivora*) or bat (*Chiroptera*) → pig/human: bite with infected saliva [71]. Pig → pig: uncommon unless infected with “furious” form and bite [73]. Pig → human: undocumented but possible [73]. Human → pig: unlikely due to behavioral factors. Human → human: only through organ/tissue transplant [72].	Canine (*Carnivora*) and bat (*Chiroptera*) species are natural hosts [71].
Toscana (TOSV); *Phlebovirus; Bunyaviridae*.	Vertebrate → sandfly (*Phlebotomus*): vector‐borne → pig/human [68, 179, 180].	Vector reservoir is sandfly (*Phlebotomus* species). Unknown vertebrate reservoir host but likely multiple livestock and wildlife species. Unclear contribution of pigs in epidemiology [179, 180].
Venezuelan equine encephalitis (VEEV); *Alphavirus; Togaviridae*.	Horse or rodent → mosquito (*Ochlerotatus* or *Culex* species): vector‐borne [70]. Mosquito → pig/human: vector‐borne [69, 70] Mosquito → human → mosquito: possible humans can develop sufficient viremia to infect mosquito [181]. Human → human: airborne or oronasal contact possible but unproven [182].	Horses are amplification host for epidemic subtypes, and rodents are reservoirs for endemic subtypes [70].
Reverse zoonotic viruses		
Norovirus (NoV); *Norovirus; Caliciviridae*.	Human → human: depending on strain fecal‐oral, vomit‐oral, food‐/water‐borne (dependent on strain) (reviewed in 86). Human → pig: possibly fecal‐oral, but not directly detected [84, 183, 184]. Pig → pig: fecal‐oral [83].	Unknown source of novel strains emerging in human populations but immunocompromised patients in nosomical settings are significant reservoirs [86].
Severe acute respiratory syndrome related‐coronavirus (SARSr‐CoV); *Betacoronavirus; Coronaviridae*.	Horseshoe bat (*Rhinolophus* species) → (unknown mammalian intermediary, possible recombination with pangolin‐CoV) → human: oronasal contact with infected secretions or excretions [26, 75, 185, 186]. Human → human: airborne [187]. Human → pig: foodborne via contaminated animal feed (restaurant leftovers) [76], possibly airborne/oronasal contact [78].	Horseshoe bat (*Rhinolophus* species) are natural hosts [185]. Humans are reservoir hosts [75].
Swine vesicular disease (SVDV); *Enterovirus; Picornaviridae*.	Human → pig: possibly fecal‐oral or oronasal contact with infected oronasal secretions or contaminated environment containing recombinant coxsackievirus B (CV‐B) and CV‐A9 [79, 80, 81]. Pig → pig: oronasal contact with environmental contamination during transportation [188].	Humans are reservoir hosts for ancestral strain [80]. Virulence decreased through subsequent passages in pigs [81, 189].
Bidirectionally transmitted viruses		
Hepatitis E (HEV); *Orthohepevirus*; *Hepeviridae*.	Pig → human: foodborne, consumption of raw or undercooked pig products, or direct contact [102, 103]. Human → human: fecal‐oral via consumption of feces‐contaminated water (type 1 and 2 in developing countries), or blood transfusion [102, 103]. Pig → pig: fecal‐oral [103]. Human → pig: undetected but possible [104, 105].	Pigs [102].
Influenza A (IAV); *Alphainfluenzavirus*; *Orthomyxoviridae*.	Human ↔ pig: airborne or oronasal contact with infectious oronasal secretions [190]. Human → human: airborne or oronasal contact with infectious oronasal secretions [190]. Pig → pig: airborne or oronasal contact with infectious oronasal secretions [190].	Wild aquatic birds are natural hosts [191]. IAV subtypes circulate in human and pig populations [12].
Influenza C (ICV); *Gammainfluenzavirus; Orthomyxoviridae*.	Human ↔ pig: possible but unknown if ICV transmitted from pigs to humans or from humans to pigs [111, 192]. Human → human: airborne or oronasal contact with infectious oronasal secretions [192]. Pig → pig: airborne or oronasal contact with infectious oronasal secretions, demonstrated in contact pigs experimentally infected with human and pig‐derived ICV [113].	Humans [192].
Picobirnavirus (PBV); *Picobirnavirus; Picobirnaviridae*.	Human ↔ pig: fecal‐oral or oronasal contact with infected respiratory secretions [193, 194].	Prokaryotes in host microbiome are likely hosts [98].
Ross River (RRV); *Alphavirus; Togaviridae*.	Marsupial or horse → mosquito (*Ades* and *Culex* species): vector‐borne. Mosquito → human/pig: vector‐borne [195]. Human → mosquito → human: vector‐borne, occurs during urban epidemics [115, 117]. Human/pig → mosquito → human/pig: possibly vector‐borne [116, 117, 196].	Marsupials in Australia [197] or horses in South Pacific islands [196].
Rotavirus genogroup A (RVA); *Rotavirus; Reoviridae*.	Human ↔ pig: fecal‐oral, respiratory, food/water‐borne [108, 198, 199, 200].	Diverse animal reservoirs including humans, porcine, bovine, ovine, pteropine, rodent, avian, and insectivore species [198, 200].
Torque teno (TTV); *Alphatorquevirus* (huTTV), *Iotatorquevirus* (TTSuV1), *Kappatorquevirus* (TTSuVK2); *Anelloviridae*.	Human ↔ pig: contact with environmental contamination, e.g., contamination of TTSuV detected in veterinary vaccines, human drugs and pork products [92, 93], and TTV found ubiquitously in the environment including water sources and hospitals [91, 94].	Unknown sources of emergent strains.

BOX 1 **Definitions of viral transmission and reservoirs used in this review.**

**Table:** 

Zoonotic viruses amplify in pigs and shed sufficient amounts to infect close contact humans, but viruses infecting humans are unable to infect pigs, thereby, pigs are viral reservoirs for humans (pig‐to‐human transmission), or zoonotic viruses infect humans directly from another reservoir species without significant involvement of pigs.
Reverse zoonotic viruses amplify in humans and transmit to pigs, but pigs are unable to infect humans in return, in which case, humans are viral reservoirs for pigs (human‐to‐pig transmission).
Bi‐directional zoonotic viruses are exchanged between humans and pigs, whereby, both hosts are reservoirs for the other (both zoonotic and reverse zoonotic).

The list of viruses shared by humans and pigs was taken from a recent study by Johnson et al., 2020 [6]. However, we were unable to find documentation of natural infection (either detection of viral genetic material or serological evidence of an antibody response against viral infection) in pigs for Ilheus, Ljungan, Monkeypox (experimental inoculation in pig skin only [29]), and Wesselsbron viruses (one study indicated serological evidence of infection in pigs but was inaccessible [30]). Tioman virus was included, despite undetected natural infection in pigs, due to evidence from an *in vivo* experimental infection study [31].

## RESULTS AND DISCUSSION

### Pigs as reservoirs for zoonotic viruses

**Fig. 1:**
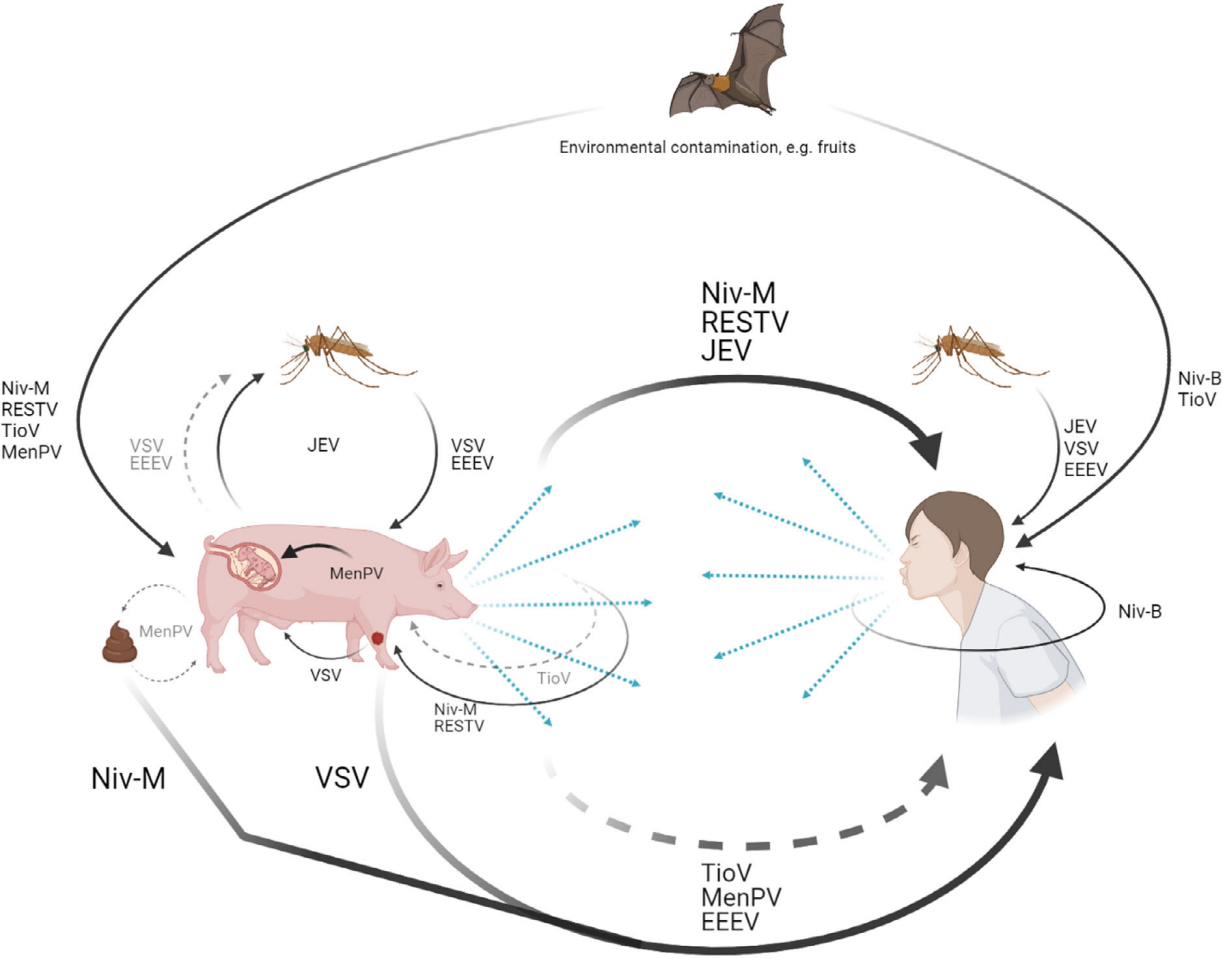
Transmission routes for seven zoonotic viruses. Solid arrows indicate transmission route, while dashed arrows indicate potential transmission route. The figure was created with BioRender.com.

The majority of the reviewed zoonotic viruses originate from wildlife reservoirs (Table 1). Pigs are significant intermediate and amplification hosts for the transmission of at least seven wildlife viruses to humans: Nipah (NiV), Japanese encephalitis (JEV), Eastern equine encephalitis (EEEV), Vesicular stomatitis (VSV), Reston ebola (RESTV), Menangle (MenPV), and potentially Tioman (TioV) (Table 1). Transmission routes of these zoonotic viruses from pigs to humans are illustrated in Fig. 1, which are generally linked to occupational exposure.

Global livestock abundance and destruction of wildlife habitats have been associated with increased zoonotic spillover risk [6]. Following a rapid increase in the past few decades, approximately 800 million to 1 billion pigs are produced globally each year in often dense and genetically homogenous populations [32, 33], owing to 95% of genetic resources being exported from Europe and the USA to developing countries between 1990 and 2005 [34]. Although increased homogeneity in a swine herd is unlikely to increase their susceptibility to epidemics, the severity of epidemics is likely to be enhanced [35]. Furthermore, the frequency of animal turnover with immunologically naïve litters of piglets in swine herds can stunt the development of herd immunity against viral infections and enable viral persistence [36].

Deforestation and encroachment of pig farms into *Pteropus* fruit bat species habitats have been implicated in causing the zoonotic NiV epidemic in pigs and human pig farm workers in Malaysia and Singapore in 1999 [37]. The spillover of NiV‐Malaysia (NiV‐M) into pig herds was traced back to two introductions from fruit bats, with isolates from local bats, pigs, and humans sharing >99% nucleotide homology [10, 38, 39], indicating transmission between hosts required limited viral adaptation. However, humans developed more severe disease with 40% case fatality rate compared to 1‐5% in pigs [40]. This difference in disease severity could be linked to higher expression of the receptor ephrin‐B2 on human tracheal and bronchial airway epithelial cells than in pigs, leading to more efficient infection [41]. NiV‐M did not transmit between humans and viral RNA was isolated from 30% of infected throat swabs [42]; therefore, it seems unlikely that infected humans posed a risk to pigs.

Pigs contribute to the epidemiology of three zoonotic arthropod vector‐transmitted viruses: EEEV, JEV, and VSV. In addition to causing viremia in pigs [43, 44, 45], EEEV can be recovered from oropharyngeal, rectal, and tonsil swabs, JEV can shed in oronasal secretions, and VSV can exude from ruptured vesicular fluids, providing further transmission routes to close contact humans (Fig. 1) [43, 47, 48, 49, 50]. However, VSV has infrequently infected farm and laboratory workers [51], likely due to the capability of human myxovirus resistance protein dynamin‐like GTPase 1 (M×A) in reducing VSV replication by 90% compared to the porcine homolog Mx1, which inhibits only 25% of VSV replication [52, 53, 54].

Antibodies against RESTV were detected in 6.3% of exposed pig farm workers in the Philippines [55]. Unlike other ebolavirus species, which cause severe hemorrhagic fever in humans [56], RESTV is unable to suppress interferon (IFN) signaling immune response in humans [57]. However, pigs develop gross abnormalities in the lymphatic and respiratory systems after experimental infection and shed RESTV in nasopharyngeal secretions, which transmit RESTV to neighboring pigs [58].

An outbreak of MenPV occurred in an Australian piggery farm in 1997 with symptoms of reproductive disease in pigs, which included increased fetal death and abnormalities, and stillborn piglets [59]. Additionally, neutralizing antibodies were detected in adult pigs and two farm workers who developed an unexplained febrile illness [59, 60]. MenPV isolated from a stillborn piglet replicated in secondary lymphoid organs and intestines in experimentally infected pigs and shed in oronasal secretions, feces, and urine for under a week [61]. The source of MenPV was assumed to be local *Pteropus* fruit bat species based on serological evidence and later confirmed following the isolation of MenPV from fruit bat urine samples, which shared 94% nucleotide homology to the pig isolates [59, 62].

TioV was also discovered in *Pteropus* fruit bat species in Tioman Island, Malaysia [63]. Outbreaks of TioV have not been reported in either humans or pigs, but due to fruit bats harboring other zoonotic viruses, a serological survey of the Tioman Island population found 1.8% of islanders were seropositive for antibodies against TioV [64]. TioV is unable to inhibit IFN‐α/β signaling in human kidney cells, but can interfere with proinflammatory cytokine interleukin 6 (IL‐6) and IFN‐β promoter induction to cause infection [65]. Following experimental infection in pigs, TioV was isolated from oral swabs and neutralizing antibodies developed without inducing clinical signs [31]. This implicates pigs as potential amplification hosts if TioV spills over from bats.

### Other reservoir host species for zoonotic viruses

Pigs appear to be minor, incidental hosts in the transmission chain for eleven zoonotic viruses. Although, more research is required to substantiate the insignificant contribution from pigs in the maintenance of many of these viruses. The majority are vector‐borne viruses: Toscana (TOSV), Venezuelan equine encephalitis (VEEV), Banna, Cache Valley, Chandipura, Getah, and Louping ill, and three are non‐vector‐borne viruses: rabies (RABV), encephalomyocarditis, and foot‐and‐mouth disease virus (Table 1).

Despite causing acute meningitis in humans [66, 67], the reservoir host species maintaining TOSV remains unknown, but likely involves a cyclic combination of arthropod, wildlife, and domesticated animals, akin to most other arbovirus maintenance cycles (Table 1). One serological survey detected IgG antibodies against TOSV in 22% of tested pigs in Spain [68], but further research efforts in pigs are lacking. Serological surveys for VEEV infection in pigs have also received limited attention since the last survey conducted in 1971 [69]. However, horses and rodents have been identified as the main amplifying hosts for epidemic and endemic strains of VEEV [70].

Other zoonotic viruses present a threat to the wider human population, beyond immediate farm and laboratory workers. Each year, RABV causes 59,000 deaths in humans usually bitten by rabid canines or bats [71]. Although RABV has been isolated from human secretions, the risk of human‐to‐human transmission is almost exclusively through organ transplantations [72]. RABV incidence in pigs is rare, and the “furious” form causing aggression with biting has only been recorded once in China [73]. As a generalist virus capable of infecting a wide range of species, RABV genetic diversity correlates with geographical origin rather than specialization in different host species, as RABV isolated from a pig shared 99.7% nucleotide homology in the partial N gene to a circulating “street” strain from a rabid canine isolated in the previous year [73].

### Humans as reservoirs for reverse zoonotic viruses

**Fig. 2:**
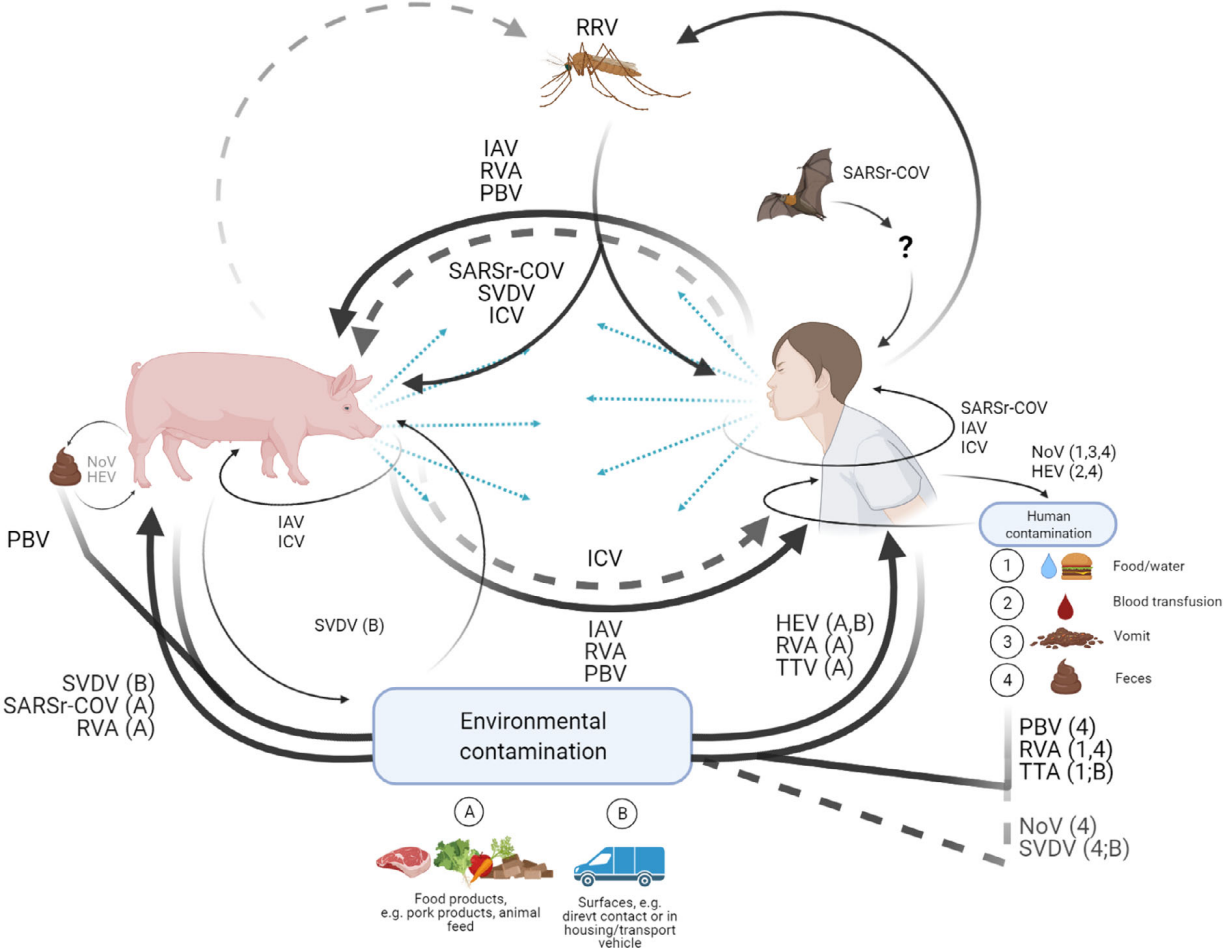
Transmission routes for three reverse zoonotic and seven bidirectionally transmitted viruses. Solid arrows indicate transmission route, while dashed arrows indicate potential transmission route. The figure was created with BioRender.com.

Humans have spread three viruses: severe acute respiratory syndrome‐related coronaviruses (SARSr‐CoV), swine vesicular disease (SVDV), and noroviruses (NoV), to pigs through varied transmission routes (Table 1) illustrated in Fig. 2 together with bidirectionally transmitted viruses (addressed in the next section).

Although SARSr‐CoV originate from *Rhinolophus* horseshoe bat species and spilled over into humans through an intermediary species, humans rapidly became an effective transmitting host and viral reservoir for SARS‐CoV in 2003 and SARS‐CoV‐2 in 2019 [74, 75]. SARS‐CoV was transmitted to pigs in China presumably via contaminated feed from restaurant leftovers [76], but there has been no evidence of natural infection in swine with SARS‐CoV‐2. However, both SARSr‐CoV appear to replicate poorly in pigs [77, 78], possibly due to less efficient viral attachment to the porcine angiotensin‐converting enzyme 2 (ACE2) homolog receptor, which shares 81% nucleotide identity with the human ACE2 receptor [75, 78].

During human meningitis epidemics between 1948 and 1964, SVDV emerged in pigs as a genetic sublineage of human‐infecting coxsackievirus B (CV‐B) [79, 80, 81]. Periodic outbreaks in pigs arose in Europe and Asia until 2007 with SVDV becoming progressively adapted to swine as later SVDV isolates (post‐1990s) lost the ability to bind human decay‐accelerating factor as a co‐receptor and infect humans [82].

Highly genetically diverse NoV infect a broad range of species, but strains belonging to genogroup II (GII) exclusively infect humans and pigs [83]. Human‐associated NoV (huNoV) have been detected in pigs, but porcine‐associated NoV (porNoV) have never been detected in humans [84, 85, 86]. porNoV were unable to bind histo‐blood group antigens (HBGA) as co‐receptors on human cells, whereas huNoV‐GII.P4 was able to bind to duodenal and buccal tissues from either A+ or H+ phenotype HBGA pigs [84, 87].

### Bidirectional viral transmission

Theoretically, a virus with the ability to infect and induce viral shedding in both humans and pigs can transmit between the two species. Non‐enveloped viruses are typically stable in the environment, which increases potential routes for transmission [88, 89, 90]. Seven viruses demonstrate bidirectional transmission by this principal (Table 1 and Fig. 2), four of which are non‐enveloped: Torque teno (TTV), picobirnavirus (PBV), hepatitis E (HEV), rotavirus A (RVA), and three are enveloped: influenza A (IAV), influenza C (ICV), and Ross River (RRV).

TTV and PBV are considered opportunistic pathogens due to their ubiquitous detection in both diseased and healthy human and pig populations and in various environments [91, 92, 93, 94, 95, 96]. Although specific TTV species of varying genome sizes are associated with human or pig infection, human‐associated *Alphatorquevirus* TTV species (huTTV) have been detected in 80% of pig sera samples and porcine‐associated *Iotatorquevirus* and *Kappatorquevirus* TTV species (TTSuV1 and TTSuVK2) have been detected in 92.5% of human sera samples [97], indicating viral exchange between the hosts. Growing evidence indicates PBV infects prokaryotes in the microbiome of humans and pigs [98]. Nevertheless, a genetic association between PBV isolated from humans and pigs has been suggested [99, 100, 101].

Humans are typically infected with HEV following the consumption of raw or undercooked pork products in developed countries and through the fecal‐oral transmission route in developing countries via consumption of water contaminated with human feces [102, 103]. Viremia peaks during the incubation period and the early symptomatic phase, with viral shedding in feces [102, 103]. While pigs are significant sources of HEV for humans, experimental infection in pigs with HEV isolated from humans has also been demonstrated [104, 105].

Similar to NoV, RVA attaches to HBGAs as co‐receptors to infect host cells, the phenotype of which depends on the VP8 domain of protease‐cleaved protein (P) types rather than the host species [106]. Unlike NoV, however, reassortant viruses with segments of human RVA origin have been found in pigs and vice versa [107, 108].

The exchange of IAV between humans and pigs is well known. Reassortant IAV generated with segments originating from human and swine IAV have been found in both host populations [12]. One high profile example was the novel genotype of H1N1 virus, which caused a human pandemic in 2009 after a quadruple reassortant IAV containing segments from avian IAV, human H3N2 subtype, Eurasian avian‐like swine IAV, and classical swine H1N1 subtype jumped from pigs into humans and back into pigs [109, 110].

Although humans were the only known natural host for ICV [111, 112], ICV has also been isolated from naturally infected pigs [109]. ICV strains isolated from humans during 1988‐1990 were highly related to the swine isolates obtained in China during 1981–1982 [111, 113], strongly suggesting interspecies transmission between humans and pigs; although, it is unknown whether the virus had transmitted from pigs to humans or from humans to pigs [111]. There is increasing evidence that other influenza species (influenza B and influenza D) are able to infect both humans and pigs and transmit between the two hosts [114].

Unlike all other zoonotic arboviruses in Table 1, RRV can potentially transmit between humans and pigs via mosquitoes. Human‐to‐mosquito‐to‐human transmission has been demonstrated during urban epidemics and pigs can also develop viremia, albeit at lower viral titers than humans [115, 116, 117].

### Viral emergence, molecular evolution, and generation of diversity

To spill over into human or pig populations, either viruses possess intrinsic ability to pass through epidemiological barriers when the permitting factors align (without significant alteration to the viral genome) or viruses must first undergo substantial genetic changes to infect new host cells and evade host immune responses. Genetic divergence is driven by mutation, recombination, and reassortment and the resulting variants, haplotypes, or reassortants either propagate or diminish by various selective processes as the virus adapts to the new host [118, 119].

RNA viruses are exceedingly more likely to be zoonotic than DNA viruses [120], given their high nucleotide substitution rates of approximately 1 × 10 ^−3^ nucleotide substitutions per site per year (ns/s/y) on average and rapid ability to adapt [121]. This is reflected in our review as all except one virus encode an RNA genome (Table S2). Nucleotide substitutions in most viruses with RNA genomes occur during replication by error‐prone, viral‐encoded RNA polymerases, while viruses with DNA genomes employ the host cell DNA polymerase with exonuclease activity to correct errors and are additionally subjected to post‐replication repair systems [119, 122]. However, TTV has a DNA genome with a comparable mutation rate to RNA viruses (0.53‐0.55 × 10^‐3^ ns/s/y [123]) and is highly genetically diverse, which could be attributed to the persistent nature of TTV infections in the host [124].

Nucleotide substitution rates and the number of susceptible host species are uncorrelated across the reviewed viruses (Table S2). Vector‐borne RNA viruses generally exhibit significantly lower mutation rates than non‐vector‐borne RNA viruses, with highly genetically similar strains infecting wide ranges of hosts (Table S2). For non‐vector‐borne RNA viruses, it is plausible that maintaining high mutation rates is necessary to adapt to a wide range of hosts. Encephalomyocarditis and foot‐and‐mouth disease viruses infect a broad range of hosts (30 and 72 documented hosts, respectively) and exhibit significantly higher mutation rates (1.61 and 1.45 × 10 ^−3^ ns/s/y, respectively) than vector‐borne viruses [6, 121, 125]. However, the number of infected hosts is not a reliable proxy for mutation rate; Chandipura virus (CHPV) has a host range of 6 and the highest mutation rate at 6.577 × 10 ^−3^ ns/s/y, RABV has the widest host range (126 known hosts) but a lower mutational rate (0.09 × 10 ^−3^ ns/s/y), and SVDV rapidly adapted to swine after introduction from humans (3.84 × 10^‐3^ ns/s/y) (Table S2). Instead, mutation rates are more likely influenced by the efficiency of virus–host cell interactions, host immune evasion, and viral reproductive strategies, among many other biotic and abiotic factors.

Major genetic changes in viruses can occur by recombination and reassortment events when host cells are co‐infected with at least two viral strains (variants or distant relatives), which interact during replication to form progeny with genetic material from both strains [118, 119]. In general, recombination is prevalent in single‐stranded, positive‐sense RNA viruses with the exception of Flaviviruses where recombination is rarely observed [118]. Novel SVDV emerged in pigs because of a probable recombination event between human‐infecting coxsackievirus B (CV‐B) and CV‐A9; although, it is unknown whether the recombination event occurred in pigs or humans [80]. Polymerase (P) types of human‐ and pig‐associated NoV frequently recombine with common breakpoints between open reading frame junctions [126, 127, 128, 129, 130], but such recombinants have only been detected in pigs [85]. Even though single‐stranded, negative sense RNA viruses in general show lower rates of recombination, reassortment is frequently observed in Orthomyxoviridae, such as influenza A virus, which belong to the single‐stranded, negative sense RNA viruses. Reassortment is restricted to segmented RNA viruses and can result in rapid genetic change by formation of reassortants with novel genome combinations [118]. Twenty‐five percent of the assessed viruses in this review have a segmented genome, potentially making these viruses more disposed to fast adaptation to a new host/interspecies transmission.

### Challenges in determining viral transmission

Our assessment of viral transmission is based on past strains of viruses. The viral landscape is under constant selective pressures, and the rapid and continuous generation of extensive genetic diversity is challenging to anticipate. Emergence of novel antigenic variants of viruses can undermine vaccination efforts, and vaccine availability against the majority of viruses is low (Table S2). Identifying the host factors a virus would need to adapt to is one modeling strategy to predict future variants, e.g., identifying viral–host protein interactions between the protein homologs in different hosts or the use of alternative host cell receptors.

RESTV is currently non‐pathogenic to humans, but substitutions of three amino acids in RESTV VP24 protein might enable binding to human karyopherin alpha5, which block innate immunity pathways in the same manner as other related pathogenic ebolaviruses [57, 131, 132]. In addition, a truncation in RESTV VP30 in a fraction of the RESTV isolates from pigs is characteristic of the Zaire ebolavirus adaptation to human cells during several months of human‐to‐human transmission in the 2013‐2016 ebolavirus disease outbreak [133].

Alternatively, wildlife viruses may attenuate as they passage through swine herds. NiV‐M, which was transmitted from bats‐to‐pigs‐to‐humans, caused a 40% case fatality rate in humans, while NiV‐Bangladesh genotype was transmitted directly from bats‐to‐humans via contaminated date palm sap causing over 70% case fatalities and has even transmitted onward to first contact humans [134]. The nucleotide difference between the two genotypes (8.2% [39]) is the most likely explanation for the difference in case fatality rates. Thus, viral attenuation through nucleotide changes in an intermediary host is a potential outcome.

Interactions between viruses and bacteria in the host microbiome may be another hidden factor facilitating viral transmission between humans and pigs. Certain bacteria express HBGAs to facilitate attachment of NoV to B cells, and *CagA*‐positive *Helicobacter pylori* induces HBGA expression in the mucosa of individuals without a functional FUT2 gene and HGBA phenotype [135, 136]. This can potentially increase the replication efficiency of particular NoV and RVA genotypes infecting humans and pigs.

Routine surveillance programs have been established for only some viruses in pigs (e.g., IAV [137]), and a few others are notifiable to international health bodies upon detection [138]. Many outbreaks lack real‐time monitoring and sampling in swine herds and humans, which can make retrospective analyses difficult and viral records incomplete (e.g., SARS‐CoV‐2 [26]). The choice of screening assays may also exclude some viruses. However, recent technical developments of next‐generation sequencing or probe‐based techniques with high‐throughput capabilities allow characterizing entire viromes of large populations a viable option. The overall aim of surveillance programs for emerging pathogens and zoonosis should be to act as early detection/warning systems because the success of limiting the spread of, e.g., a new zoonotic virus to a great extent relies on the possibility to contain it *before* it jumps to the first human. This in turn calls for more basic research into identification of reliable viral and host markers of species specificity for the different types of viruses combined with a *One Health*‐oriented design of the monitoring programs, i.e., by the inclusion of more targeted sampling of people in close contact with animals, e.g., swine.

Experimental studies involving human volunteers are rare. Only IAV, ICV, NoV, and RVA have been administered in challenge studies, usually with human‐derived isolates, common circulating genotypes in the population, or attenuated viral strains [139, 140, 141, 142]. Therefore, experiments with viruses to study human‐related dynamics rely on cell culture, explants, or animal models, which have some restrictions for application in a human population. Nevertheless, these experiments provide valuable data, particularly concerning specific virus–cell interactions.

## CONCLUDING REMARKS

The list of 27 viruses shared by humans and pigs are generally regarded as zoonotic [6]. Reverse zoonosis or humans’ ability to transmit viruses to other animals is overlooked in some cases [8]. This review gathered evidence to assess the direction of viral transmission in the context of humans and pigs. Where direct detection was lacking, we theorized whether the virus could infect and transmit to the other host based on viral entry requirements, ability to establish infection, activation of immune responses, and shed in transmissible routes.

Transmission routes and viral sources are illustrated in Figs 1 and 2. Pigs are or have potential to be significant reservoirs, intermediaries, and amplifiers for at least seven zoonotic viruses; humans have been the source of three reverse zoonotic viruses in pigs; and humans and pigs possibly exchange seven viruses back and forth (Table 1).

The authors thank the FluZooMark group for contributing to discussions of the content of the review.

## CONFLICT OF INTEREST

The authors have no conflicts of interest to declare.

## Funding information

The work presented in this review is part of the FluZooMark project supported by Novo Nordisk Foundation (grant NNF19OC0056326).

## Supporting information


**Table S1.** Framework of viral factors with associated relevance and assumptions considered in the review.

**Table S2.** Highlights of collected data based on the framework of factors in Table S1, which is used to inform viral transmission direction in Table 1. Distinctions are drawn between humans and pigs where appropriate.
