# Supplementary material for: Zoonotic and reverse zoonotic transmission of viruses between humans and pigs
Source: APMIS. 2021 Oct 18;129(12):675–93. doi: 10.1111/apm.13178 (PMC9297979; doi:10.1111/apm.13178)
Supplement: Supplementary file 1 — Table S1. Framework of viral factors with associated relevance and assumptions considered in the review. [file APM-129-675-s002.docx]

**6. Supplementary material**

Supplementary Table 1. Framework of viral factors with associated relevance and assumptions considered in the review.

| **Viral factor** | **Relevance and assumptions** |
| --- | --- |
| Virion structure (enveloped or non-enveloped) | Determines viral-cell fusion and maturation pathways; suggests environmental stability and transmission route. |
| Genome | Indicates replication mechanism and generation of genetic variation e.g. reassortment in segmented viruses. |
| Infectivity | Detection or isolation of the viral genome or infectious particles demonstrates viral infection (without vaccination). Serological evidence of antibody response without viral detection implies past clearance or suppression of viral infection. |
| Experimental infection | Provides evidence of cross-species infectivity if viral isolate from one host can infect the other host (including cell culture or explants). |
| Occurrence | High occurrence in the population might reveal the host as a possible reservoir. Outbreaks in one host associated with infections in the other might signal direct transmission. |
| Phylogenetic relationships, genetic diversity, and molecular evolution (mutation rate) | Degrees of sequence homology and phylogenetic relatedness between isolates from hosts can illustrate common ancestry, progressive host adaptation, species-specific strains, genetic exchange between strains, single or multiple introductions to population, or geographical or temporal effects. |
| Clinical signs | Symptoms can identify affected organs, severity of infection, and transmission route. Asymptomatic infections imply possible undetected cases. |
| Attachment and entry to host cells | Availability of receptor and co-factors dictates infection ability and efficiency. |
| Pathogenesis | Explains viral disease mechanisms, identifies target cells, organs, and barriers to viral spread. |
| Shedding | Displays ability to transmit virus and transmission route. |
| Immune response | Reveals infection initiation and capability of host to control infection. |
| Immune evasion | Avoid and interfere with the host`s immune system allows infection to progress. |
| Vaccination | Preparedness for outbreaks and potentially provides selective pressure for immune escape mutants. |
| Interactions with other viruses or bacteria | Immunosuppression by other viruses or bacteria may enable infection or worsen severity of other infections or microbiomes may assist or prevent infection. |

Supplementary Table 2 (attached in separate file). Highlights of collected data based on the framework of factors in Supplementary Table 1, which is used to inform viral transmission direction in Table 1. Distinctions are drawn between humans and pigs where appropriate.
